# Photolysis for the Removal and Transformation of Pesticide Residues During Food Processing: A State-of-the-Art Minireview

**DOI:** 10.3389/fnut.2022.888047

**Published:** 2022-05-19

**Authors:** Qian Xiao, Xiaoxu Xuan, Grzegorz Boczkaj, Joon Yong Yoon, Xun Sun

**Affiliations:** ^1^Key Laboratory of High Efficiency and Clean Mechanical Manufacture, Ministry of Education, School of Mechanical Engineering, Shandong University, Jinan, China; ^2^State Key Laboratory of Pollution Control and Resource Reuse, College of Environmental Science and Engineering, Tongji University, Shanghai, China; ^3^Department of Civil Engineering, The University of Hong Kong, Pokfulam, Hong Kong SAR, China; ^4^Department of Process Engineering and Chemical Technology, Faculty of Chemistry, Gdańsk University of Technology, Gdańsk, Poland; ^5^Department of Mechanical Engineering, Hanyang University, Ansansi, South Korea; ^6^Department of Mechanical Engineering, The University of Hong Kong, Pokfulam, Hong Kong SAR, China

**Keywords:** food processing, photolysis, pesticide residues, pollutant decontamination, transformation

## Abstract

Pesticide residues are of great significant issue that exerted adverse effects on humans. There is a need for effective and non-toxic decontamination of pesticide residues during food processing. In this minireview, the recent advances in the degradation of pesticide residues by photolysis have been firstly described during food processing. The mechanisms of pesticide residues destruction by photolysis were discussed accordingly. Finally, applications of photolysis in the degradation of pesticide residues from beverages, fresh produce, and food rinse waste were also summarized.

## Introduction

Global food supply should be enhanced by 70–100% by 2050 in order to meet the demand for the improvement in population size (1). The pesticide has been widely applied to control insects, fungus, and weeds for increased food production in the world (2, 3). Some amounts of pesticide residues could remain on foods such as vegetables and fruits that accounted for 30% of an individual’s diet, which is frequently transferred to markets and consumed by humans without proper washing or with minimal processing (4). Unfortunately, pesticides have been identified as a major problem by a variety of countries because of their persistence in environments and adverse effects on human health, including the destruction of biodiversity, and dermatological, gastrointestinal, neurological, carcinogenic, respiratory, reproductive, and endocrine effects (5–7). Therefore, it is of great significance for the degradation of pesticide residues during food processing.

Pesticide residues have been categorized as insecticides, herbicides, and other pesticides by the United States Environmental Protection Agency (US EPA) (8). Several methods have been utilized to degrade pesticide residues during food processing, including conventional techniques, and advanced treatment techniques, i.e., non-thermal physical methods (4), and chemical methods (9). Conventional techniques mainly fall into cleaning with various reagents, peeling, drying, heating, and other processes (10, 11). Chemical methods mainly include ozone (9), and photocatalytic oxidation (12–14); non-thermal physical methods primarily contain cold plasma, photolysis, electron beam, electrolyzed water, etc. (14–17). Conventional techniques, i.e., cleaning in water, had limited effects on pesticide removal based on that most pesticides are hydrophobic in nature (9, 18, 19). Cleaning and peeling, drying, concentration, fermentation, and other processes easily transform pesticides into toxic products, which showed the limited applications during food processing (9, 20). Moreover, ozone can cause negative alternations on food components: loss of some vitamins, phenolic compounds, ascorbic acids, and carotenoids, changes in color, sensory characteristics, and other adverse effects (21–23). Also, degradation products of pesticide residues after ozone treatment were demonstrated to be more toxic than the parent compounds (24, 25). These further have retarded the development of ozone-based technologies. Similarly, although photocatalytic oxidation did show high pesticide degradation performance, they significantly increased the toxicity of the treated solutions, inhibiting their further applications in food processing. This together led to the development of non-thermal physical methods.

Non-thermal physical methods showed numerous characteristics of the non-thermal treatment, economic friendliness, low costs, high efficiency, and low reaction time (4, 26). Amongst them, the photolytic techniques have been reported to be an effective, non-chemical, and residue-free approach (27–29), indicating their good prospects in food processing, such as beverages, fresh products including honey, and dairy products, and rinse wastewater. Therefore, it is necessary to comprehensively review the available and newest literature associated with pesticide degradation by photolysis. Also, this minireview article is important and useful to readers in the areas for understanding the mechanism, most updated progress, and the primary application of this technology. To the best of our knowledge, there is no comprehensive review of the application of photolysis.

In this minireview, our aim is to present recent advances in photolysis for the degradation of pesticide residues during food processing. The mechanisms of pesticide residues destruction by photolysis were discussed accordingly. Finally, applications of photolysis have been summarized in the degradation of pesticide residues in beverages, fresh products, and food rinse waste.

## The Mechanism of Photolysis for the Degradation and Transformation of Pesticide Residues

Photolysis mainly includes ultraviolet (UV) light irradiation (27, 30–34), pulsed light (PL) technology (4, 35, 36), as well as visible light illumination (37, 38). The UV spectrum falls into UV-A (380–315 nm), UV-B (315–280 nm), UV-C (280–200 nm), vacuum-UV (VUV) (200–100 nm), and extreme UV (100–1 nm). The low-pressure mercury vapor lamps emitting at 185 and 254 nm, and xenon excimer lamps emitting at 172 nm have been often utilized for applications in food processing (2). Photolysis was first proposed for the degradation of pesticide residues (i.e., organochlorine insecticides) in fluid milk and butter oil by Li and Bradley (39, 40). Since then, the development and applications of photolysis for different pesticide decontamination purposes were extensively carried out (41). Fundamentals of photolysis have been proposed in the literature.

**FIGURE 1:**
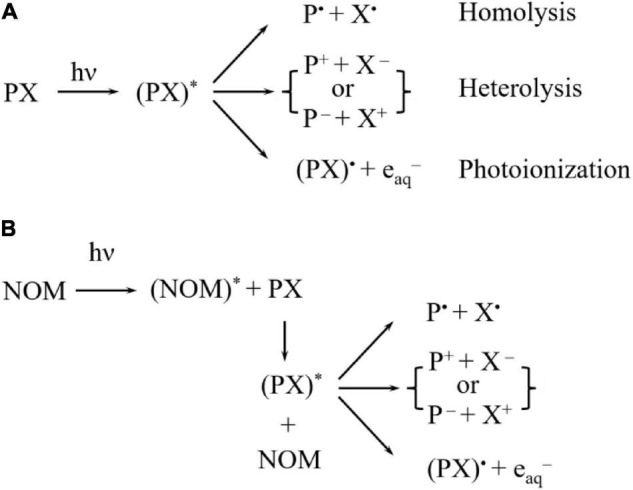
The degradation mechanism of pesticide residues by **(A)** direct and **(B)** indirect photolysis (42).

Photodegradation of pesticide residues could be achieved *via* two mechanisms: direct photolysis and indirect photolysis. On the one hand, in the direct photolysis, pesticides that absorb energy from the UV light can trigger chemical reactions upon the irradiation of UV light: the chemical structure of a pesticide can be transformed into an excited state and then into a triple-state, which would finally undergo *via* homolysis, heterolysis, and photoionization, which could be seen in Figure 1A (42). On the other hand, in indirect photolysis, sensitizers such as natural organic matters (NOM) (30) (Figure 1B), absorbing photos would result in the production of highly active species (i.e., NOM*) through the excitement of UV light, which would react with pesticide residues. Just as stated above, pesticide residues could further decompose through three main pathways including homolysis, heterolysis, and photoionization. It has been reported that immediate products were generated after photodegradation by involving structural changes such as dehalogenation, desulfuration, dealkylation, and oxidation of the alkyl chains (4), which could be identified through mass spectrometry (MS) analysis. Degradation pathways include multiple successive and competitive steps with the later destruction processes being involved in the earlier formation of degraded processes (4). In addition, solution pH could exert an effect on the formation of immediate species of pesticide residues, and the degradation pathways accordingly (43). Understanding the degradation and transformation of pesticide residues during food processing not only can help to design and optimize the decontamination process, but also provide important information for further reducing food safety risks.

## Applications of Photolysis During Food Processing

### The Decontamination of Beverages

As reported, significant degradation of patulin could be achieved in apple juice, which was attributable to its absorption of photons in the UV-C range in Table 1 (45–47). Similarly, Chandra et al., found that patulin could not be degraded in water, but would be very efficiently decomposed in apple juice at the same conditions with UV illumination as shown in Table 1 (44). The authors demonstrated that riboflavin in apple juice played a significant role in the photodegradation of patulin. This inspired other scholars to further investigate the effect of matrix components in food and their importance on the photodegradation of pesticides and metabolites. Dai et al. conducted the degradation of pesticide residues such as cartap and nereistoxin in tea beverages by photolysis in Table 1 (27). The authors reported that water-soluble components in beverages did affect the degradation of pesticide residues. As known, tea samples comprise various types of water-soluble chemical components, such as total sugar, caffeine, and tea polyphenols. Catechins with hydroxyl groups, one type of polyphenol, in green tea, could complex with cartap through amide bonds, further accelerating cartap degradation (50). Reportedly, catechins in black tea firstly transformed into theaflavin and thearubigin, then complexed with caffeine, and finally precipitated, which resulted in reduced hydroxyl groups. Accordingly, compared to green tea beverages, cartap was less affected in black tea beverages. Taking together, the water-soluble components in tea mainly played an inhibitive role in the photolytic process of pesticide residues (27). This was primarily attributable to the presence of polyphenols in tea beverages that could compete with pesticides for the light and quench free radicals, and thus slow down the photodegradation of cartap and nereistoxin.

**TABLE 1:** The degradation of pesticide residues in beverages.

Pesticides	Processes	Reaction conditions	Efficiency	References
Patulin	Ultraviolet (UV-C) irradiation	∼ 0.2 ppm patulin, UV-C dose of 0.4 J/cm^2^	69.47 (± 0.69)%	Chandra et al. (44)
Patulin	UV-C irradiation	1 ppm patulin, UV-C dose of 5.6 J/cm^2^	89%	Tikekar et al. (45)
Patulin	UV-C irradiation	1 ppm patulin, UV-C doses of 7.2 J/cm^2^	94.8% for 40 min	Zhu et al. (46)
Patulin	UV multi-wavelength emitting lamp	0.5 ppm patulin, pH 4.0, 25°C	∼100% in 60 min	Ibarz et al. (47)
Cartap	UV-light irradiation	1 ppm cartap, 200 W mercury lamp	98.5% at 2 h	Dai et al. (27)
Nereistoxin	UV-light irradiation	1 ppm nereistoxin, 200 W mercury lamp	100.0% in 0.5 h	Dai et al. (27)
Dichlorvos	Sunlight irradiation	4.5 μM dichlorvos at pH 3 and pH 7	0.040–0.064 h^–1^	Bustos et al. (48)
Dichlorvos	254 nm UV irradiation	4.5 μM dichlorvos, 0.1 Einstein/L	97% in 6 h	Bustos et al. (48)
Epicatechin (EC)	Blue light illumination at 438 nm	1 mM EC, 2.0 mW/cm^2^, in the presence of epigallocatechin gallate	57.9% in 3 h (epigallocatechin gallate), 64.5% in 3 h (gallic acid)	Huang et al. (49)

As stated before, irradiation absorption by a compound could lead to its degradation and transformation by direct photolysis (48). The degradation of cartap and nereistoxin in water and tea beverages reached 81.8–100% after 6 h with 200 W of UV illumination, which was mainly ascribed to the easy destruction of disulfide bonds of nereistoxin by UV irradiation (51). This could be evidenced by cartap and nereistoxin showing a maximum UV absorption at 190 nm with pH 7.21, and at 196 nm with pH 3.04, respectively, as shown in Table 1, substantiating their ability to absorb UV light (27). Additionally, different degradation behaviors were observed in a variety of tea beverages, i.e., green tea beverages and black tea beverages. Huang et al. found that the photodegradation of nereistoxin in green tea beverages was lower than that in black tea beverages (Table 1), which was attributable to the inhibitory effect of high concentrations of catechins in the former (49). Therefore, it is urgent to investigate and compare the decontamination of pesticide residues in realistic beverages.

### The Decontamination of Fresh Produce

Reportedly, the temperature played a minor role in the photolysis, whereas light intensity, irradiation time, types of pesticide residues, and UV light sources are important in the decomposition of pesticide residues by photolysis (52). Yuan et al. demonstrated the photodegradation of organophosphorus pesticides (OPs) in the honey medium, including coumaphos, methyl parathion, and fenitrothion under three different intensities (37). For instance, the degradation of coumaphos was 93.38, 96.52, and 97.02%, respectively, after 1 h with 250, 500, and 750 W/m^2^ sunlight irradiation. As a result, there was a positive relationship between sunlight intensity and the degradation of pesticide residues in the honey medium. Meanwhile, the longer the reaction time is, the faster the degradation of pesticide residues is and the lower the residual concentration of pesticide residues is. The decontamination efficiency of coumaphos reached 90% within 15 min, which was lower than that after 1 h (97.02%) under 750 W/m^2^ sunlight irradiation. Moreover, types of pesticide residues could also exert an effect on the degradation of pesticides. Amongst them, coumaphos exhibited the best degradation performance (90% of degradation after 15 min); the removal percentage of fenitrothion and methyl parathion reached 83.3, and 73.11%, respectively, within 1 h under 750 W/m^2^ sunlight illumination (37).

The effect of UV light sources on the degradation of pesticides was further investigated, including VUV, and UVC. Results demonstrated that VUV (185 nm) was more effective than UVC (254 nm) in the degradation of pesticides such as pyraclostrobin, boscalid, fludioxonil, and azoxystrobin under the same reaction condition (53). It was mainly due to VUV at 185 nm could produce more energetic photons. Additionally, Yang et al. investigated the removal of five typical pesticides from water by VUV/UV at both bench- and pilot-scale studies (5). Results showed that VUV/UV showed much more effective and energy-efficient than UV and all pesticides could be removed with an efficiency of >90% at a VUV fluence of 12 mJ/cm^2^. Furthermore, pilot-scale studies revealed that VUV/UV processes had a stable performance with acceptable energy consumption of 0.27–1.52 kWh/(m^3^order). As a result, photolysis showed the potential for the degradation of pesticides as an energy-efficient and high-efficiency technology for surface decontamination of fresh produce.

### Applications of Photolysis in Food Rinse Waste

Several photodegradation techniques by UV have been utilized for the degradation of pesticide residues from wastewater (27, 47). The pulsed light (PL) technology serves as a novel tool for the degradation of several herbicides in food rinse waste. As known, PL technology contains a successive repetition of short duration (325 μs) and high power flashes emitted by xenon lamps that range from ∼200 to 1,000 nm with a considerable amount of light in the short-wave UV spectrum. Baranda et al. investigated the photodegradation of several triazidic and organophosphorus pesticides in aqueous solutions by PL technology (35). The most studied pesticide residues were degraded very fastly, and their degradation was greater than 50% in a short period of time (milliseconds). However, there is a lack of studies on the toxicity of photodegradation products in the process. Considering that the PL technology (xenon flashlamp) had the characteristics of a mercury-free system, it could be therefore considered as a promising environmentally friendly photolytic approach for the decontamination of pesticide residues from rinse wastewater.

Moreover, the degradation kinetics of pesticide residues in wastewater could be fitted using a pseudo-first-order kinetics model by using UV irradiation (54, 55). Cunha and Teixeira (54) reported that the pseudo-first-order reaction rate constant for azoxystrobin, difenoconazole, and imidacloprid was in the ranges of 0.128–0.249 s^–1^, 0.019–0.048 s^–1^, and 0.129–0.266 s^–1^, respectively, in tomato rinse water by photolysis. In addition, the degradation of chlorpyrifos in water was carried out with sunlight illumination to extend the light spectrum to visible light. Results showed that the highest degradation rate was 4.2% per day in distilled water at the light intensity of 43,400 l× and 7.4% per day in lake water at the light intensity of 42, 200 l× (56). The faster degradation was achieved in natural water than distilled water (56, 57), demonstrating a greater application potential of solar irradiation. The enhanced degradation could be ascribed to the presence of components such as natural organic matters and thus greater formation of active species during the process (56). Admittedly, there was a slower degradation of malathion by UV alone than photocatalytic treatment processes including UV/H_2_O_2_, UV/TiO_2_, and UV/Fenton systems (43). However, it was interesting to find that no increase in toxicity was observed for the malathion aqueous solution with UV irradiation alone, whereas the toxicity of the malathion aqueous solution was increased sharply with photocatalytic processes (43). These findings indicate that photolysis, instead of photocatalytic treatment technologies, showed greater potential applications in food rinse wastewater in terms of both treatment efficiency and the toxicity of intermediate products. It needs to be noted that most research was carried out in bench- and pilot-scale studies. Future research should be paid more attention to improving pesticide residues degradation in industrial-scale studies.

## Conclusion

The photolytic technique, a promising water treatment technology, has attracted increasing attention due to improved performance, such as no chemical required, and more safety, and could be widely implemented for various foods decontamination purposes. In photolysis, the chemical reaction would occur when the light energy absorbed by pesticides is higher than the bond energy of a chemical bond in the pesticide molecule. The application potential of photolysis indicated that the photolytic technique could work in various complex environments, including beverages, fresh produce, and natural food rinse wastewater. Although some achievements have been made, there possess still many challenges before photolysis has been applied in food processing, primarily including large energy input, sequential maintenance required, as well as thus increased cost. Assessing health risks and socioeconomic impacts of immediate products in the degradation of pesticide residues should be extremely important future work.

## Author Contributions

QX: investigation, resources, writing – original draft, review, and editing, conceptualization, and supervision. XX, GB, and JY: writing – review and editing. XS: writing – review and editing, conceptualization, and supervision. All authors contributed to the article and approved the submitted version.

## Conflict of Interest

The authors declare that the research was conducted in the absence of any commercial or financial relationships that could be construed as a potential conflict of interest.

## Publisher’s Note

All claims expressed in this article are solely those of the authors and do not necessarily represent those of their affiliated organizations, or those of the publisher, the editors and the reviewers. Any product that may be evaluated in this article, or claim that may be made by its manufacturer, is not guaranteed or endorsed by the publisher.
